# Can 3D surgical planning and patient specific instrumentation reduce hip implant inventory? A prospective study

**DOI:** 10.1186/s41205-020-00077-2

**Published:** 2020-09-23

**Authors:** Anna Di Laura, Johann Henckel, Harry Hothi, Alister Hart

**Affiliations:** 1 The Royal National Orthopaedic Hospital, Brockley Hill, Stanmore, London HA7 4LP UK; 2 Institute of Orthopaedics and Musculoskeletal Science, University College London, London, UK

**Keywords:** Total hip Arthroplasty, Preoperative planning, Three-dimensional computerised planning, Implant cost, Implant inventory

## Abstract

**Background:**

Modern designs of joint replacements require a large inventory of components to be available during surgery. Pre-operative CT imaging aids 3D surgical planning and implant sizing, which should reduce the inventory size and enhance clinical outcome. We aimed to better understand the impact of the use of 3D surgical planning and Patient Specific Instrumentation (PSI) on hip implant inventory.

**Methods:**

An initial feasibility study of 25 consecutive cases was undertaken to assess the discrepancy between the planned component sizes and those implanted to determine whether it was possible to reduce the inventory for future cases. Following this, we performed a pilot study to investigate the effect of an optimized inventory stock on the surgical outcome: we compared a group of 20 consecutive cases (experimental) with the 25 cases in the feasibility study (control). We assessed: (1) accuracy of the 3D planning system in predicting size (%); (2) inventory size changes (%); (3) intra and post-operative complications.

**Results:**

The feasibility study showed variability within 1 size range, enabling us to safely optimize inventory stock for the pilot study. (1) 3D surgical planning correctly predicted sizes in 93% of the femoral and 89% of the acetabular cup components; (2) there was a 61% reduction in the implant inventory size; (3) we recorded good surgical outcomes with no difference between the 2 groups, and all patients had appropriately sized implants.

**Conclusions:**

3D planning is accurate in up to 95% of the cases. CT-based planning can reduce inventory size in the hospital setting potentially leading to a reduction in costs.

## Background

In the United States (US), Total Hip Arthroplasty (THA) has been targeted for cost containment by Centers for Medicare and Medicaid Services (CMS) because of its high cost per procedure and its increasing prevalence [1–4]. The implant is the most expensive supply item for joint replacement [1, 5].

Preoperative planning is an essential step in the preparation for elective surgery [6–8]. The technical goals of preoperative planning of the hip joint include restoration of femoral offsets and limb length [9, 10] as well as the restoration of the centre of rotation, all of which are dependent on implant size.

Starting from Computed Tomography (CT) scans, Three-Dimensional (3D) models of the patient anatomy are created in a virtual 3D environment. These models are then used to plan the operation, determining implant size, type and positioning in relation to a chosen standard frame of reference; the surgeon acquires valuable information regarding patient anatomy before surgery. The digital plan can be transferred to patient care by way of 3D printed personalised instruments and surgical guides, so-called Patient-Specific Instruments (PSI) [11, 12]. 3D printed, sterilised and used intraoperatively, the physical models aid the surgeon achieving optimal cup and stem sizing and positioning [13, 14].

Despite the evidence that CT - based planning (or 3D or virtual planning) is more accurate than conventional 2D radiograph templating in predicting implant size [15–18], it has not been widely adopted. Most 2D planning platforms are integrated into hospital Picture Archiving and Communication System (PACS) systems and are broadly available.

We aimed to better understand the impact of using 3D surgical planning and PSI on inventory size and surgical outcome of THA. Our primary objective was to assess the accuracy of a 3D-CT planning system. Our secondary objective was to evaluate if implant inventory sizes can be reduced with the use of the planning software.

## Methods

### Study design

We designed a prospective study involving a total of 45 consecutive patients undergoing primary cementless THA due to osteoarthritis (OA).

An initial feasibility study (with a group of 25 consecutive cases) was undertaken to assess the discrepancy between the planned component sizes and those implanted, to determine if it was possible to reduce the inventory size for future cases.

Following the feasibility study, we performed a pilot study to investigate the effect of an optimized inventory stock on surgical outcome: we compared a group of 20 consecutive cases (experimental group) with the 25 cases in the feasibility study (control group). The outcome measures were: 1) accuracy of the 3D planning system and PSI in predicting size (%); 2) inventory size changes (%) between the two groups; and 3) surgical outcome.

Data collection for the study was performed at our Institution between June 2017 and April 2018 and included patients who underwent primary THA. The surgery was performed through a posterior approach by a single consultant orthopedic surgeon who specializes in hip arthroplasty and has done more than 1000 primary and revision hip arthroplasties.

Prior to surgery, all patients underwent 3D planning with the CT-based software MyHip (Medacta, Castel San Pietro, Switzerland) to determine the size and orientation of the prosthesis most appropriate to restore native hip biomechanics. The MyHip planning system is specifically designed to assist the surgeon with implant selection and positioning and includes the intraoperative use of 3D printed patient-specific guides which can reproduce the surgical plan. 3D printed plastic models of the patient’s acetabulum, proximal femur and relative guides, manufactured using 3D printing, are sterilised for intraoperative use. Our institutional review board approved the study (SE16.020).

### The patients

We recruited a consecutive series of 45 patients undergoing primary THA for osteoarthritis. In the initial group selected for the feasibility part of the study there were 14 males and 11 females (mean age 64.36 years, range 39–81); in the experimental group of the pilot study there were 9 males and 11 females (mean age 71.65 years, range 54–79). Table 1.

**Table 1:** Patient demographics

	Feasibility study group (***n*** = 25)	Pilot study group (***n*** = 20)	***p***-value (significant if < 0.05)
Gender (male: female)	14:11	9:11	*p* = 0.55
Age at surgery (years)	64.36 (±11.80)	71.65 (±7.081)	*p* = 0.01
Indication (%)	OA (100%)	OA (100%)	NA

Data presented as ratios, percentages or means (±SD)

*OA* osteoarthritis; *NA* not applicable

According to the literature, the accuracy for size component prediction with CT planning is around 90%, achieved with a minimum of 25 patients (alpha level 0.05, power of 80%) [16, 17]. The number of patients in this study was chosen to provide a reasonable estimate of accuracy as well as reproducibility prior to starting a prospective study [19, 20].

### CT scanning protocol

All patients underwent pre-operative CT-scanning of the hip region and the knee joint according to a standard protocol. The scanning protocol is specifically designed to minimize radiation dose, while ensuring a good spatial accuracy. Image acquisition consisted of two short spiral axial scans: one including the whole pelvis (starting at least 2 cm above the iliac crests and continue to at least 10 cm below the lesser trochanter) and the proximal femur; the second including the distal part of the femur in the affected side at least 2 cm above the posterior condyles and extend down to the distal condyles towards the tibia [21].

### 3D surgical planning

The CT DICOM (Digital Imaging and Communications in Medicine) data was transferred to the MyHip 3D reconstruction planning software to create a patient specific 3D model of the pelvis and femurs of the subject. The engineers at Medacta planned the position and size of the prosthesis according to the surgeon’s preferences before he validated the final plan for each case.

MyHip software allows the operator to select a series of bony landmarks on the femur and pelvis in order to define preoperative parameters.

**Fig. 1:**
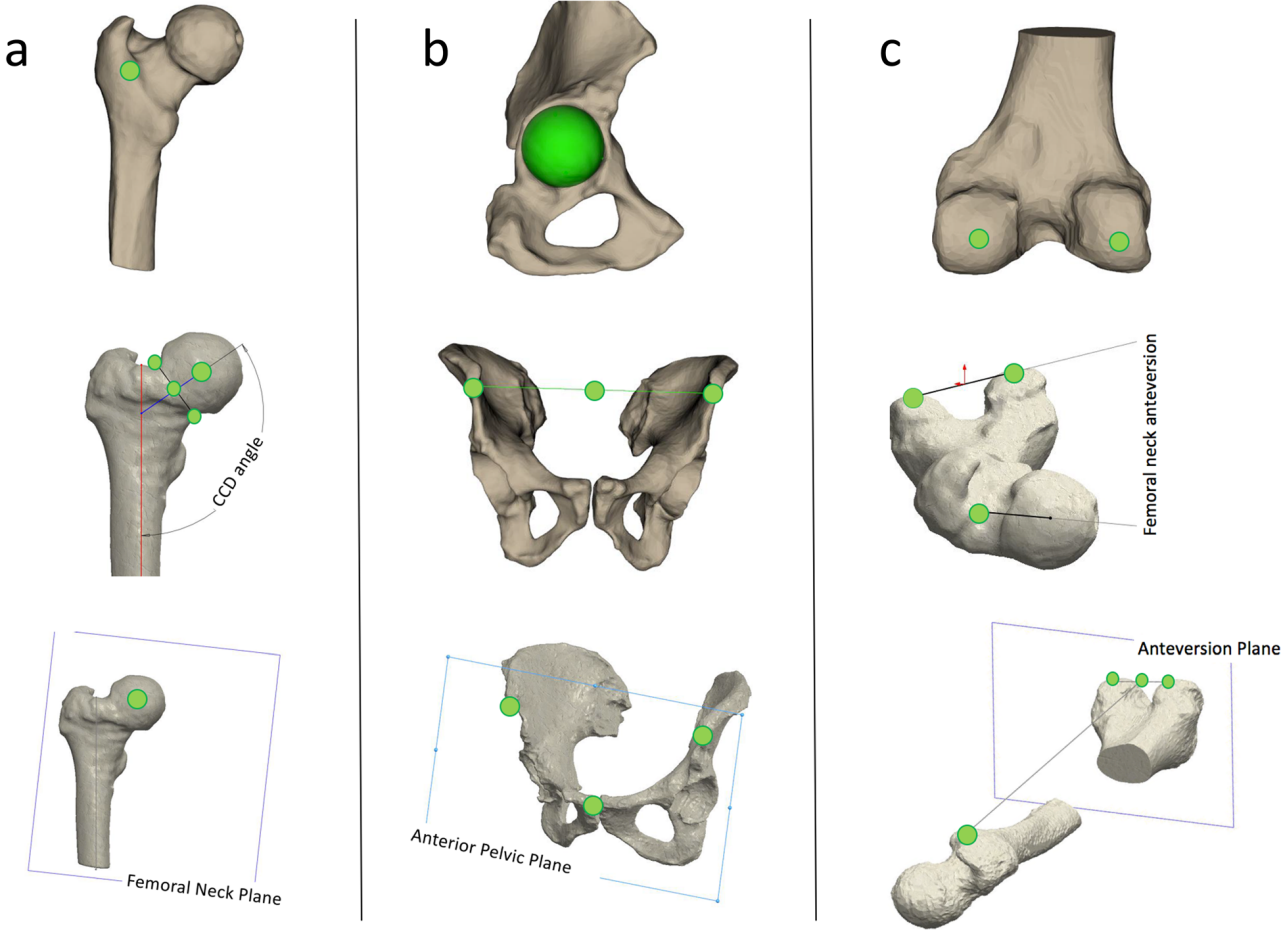
MyHip planning software. Examples of landmark selection **a** on the femur, **b** pelvis and **c** knee for the acquisition of the relevant anatomical and functional axes and planes

Nine reference points are taken on the femoral side (diaphysis section center, piriformis fossa, anterior tubercle, lesser trochanter, intertrochanteric crest, femoral neck section, center of the femoral head, medial and lateral posterior condyles). Three references are taken on the acetabular side (the acetabular cavity by fitting a sphere, the tear drop and the acetabular north). The relevant axes (femoral anatomical axis, femoral neck axis) and planes (femoral neck plane, femoral anteversion plane, anterior pelvic plane and neck cut plane) are defined by the software after anatomical landmarks acquisition; Fig. 1**.**

**Fig. 2:**
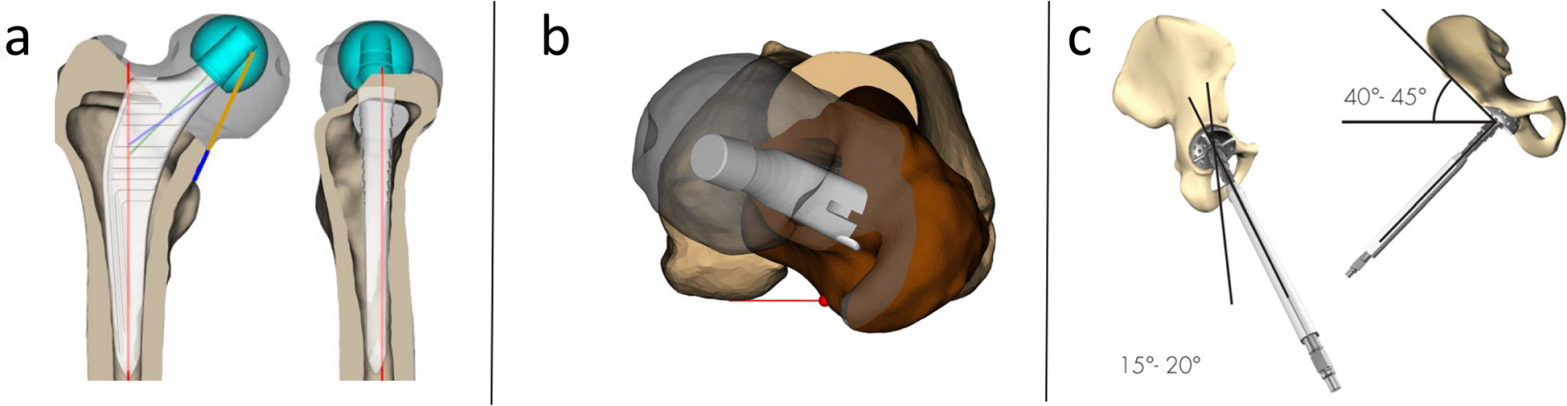
Examples of images from the MyHip software: **a** Preoperative planning of the femoral component, **b** anteversion view, **c** preoperative planning of the acetabular component positioned with 45° of inclination and 15° of anteversion (Lewinnek safe zone)

Once the operator has selected all landmarks, he proceeds with adjusting the cut height. During the planning of the stem position and of the femoral head resection, all parameters related to the femoral resection in terms of cut height, cut angle and cut anteversion can be modified according to the surgeons’ planning philosophy. The stem is positioned in terms of AP (anterior/posterior) and ML (medial/lateral) offset from the default position, Fig. 2a.

The stem size is chosen to achieve a good fit in the intramedullary canal and head size is selected to adjust the femoral offset. Stem version (Fig. 2b), leg length adjustment, cup positioning can all be adjusted to achieve optimal alignment between the two legs (using the lesser trochanter as a reference). The surgeon defines the acetabular reaming depth and the acetabular angles (anteversion and inclination). Different spatial views are available, the operator can change the size of component among those available, while adjusting the level of fit-and-fill, Fig. 2**.**

**Fig. 3:**
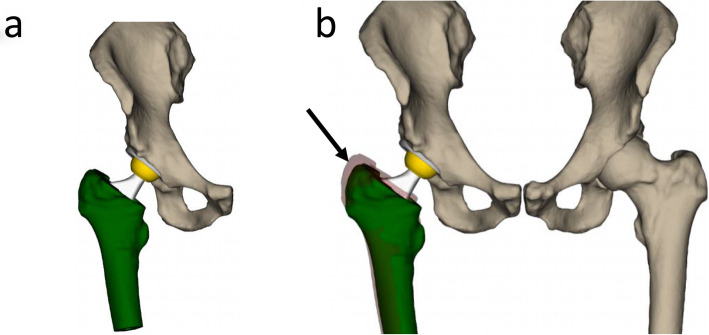
Example of (**a**) the global view and (**b**) leg length view after planning. While the first displays the affected side, the second one involves the contralateral side too. This view is used to check the alignment between the two femurs, using the lesser trochanter as reference. The red model (arrow) represents the pre-op position and the green is the post-op one

The result of the previous steps can be evaluated in the so-called global view and the leg length view. While the first one is focused on the affected side, the second one involves the contralateral side for comparison. This view is used to check the alignment between the two femurs, using the lesser trochanter as reference, Fig. 3**.**

A single, cementless implant system was used (Quadra-H System femoral components and Mpact System acetabular components, Medacta, Switzerland).

Surgical guides and anatomical models were produced by Selective Laser Sintering technology (SLS). PA 2200, a type of nylon that is a special moulding material developed by German EOS for their SLS rapid prototyping equipment, was used for 3D printing. (EOS, Munich, Germany). The components were sterilised either using gamma irradiation and delivered to our hospital or autoclaved at the hospital before intra-operative use.

### Intra-operative assessment and use of PSI

For each patient, the planned sizes of acetabular and femoral components were recorded and compared to the actual sizes used at surgery. Complications such as intra-operative femoral fracture was also recorded.

**Fig. 4:**
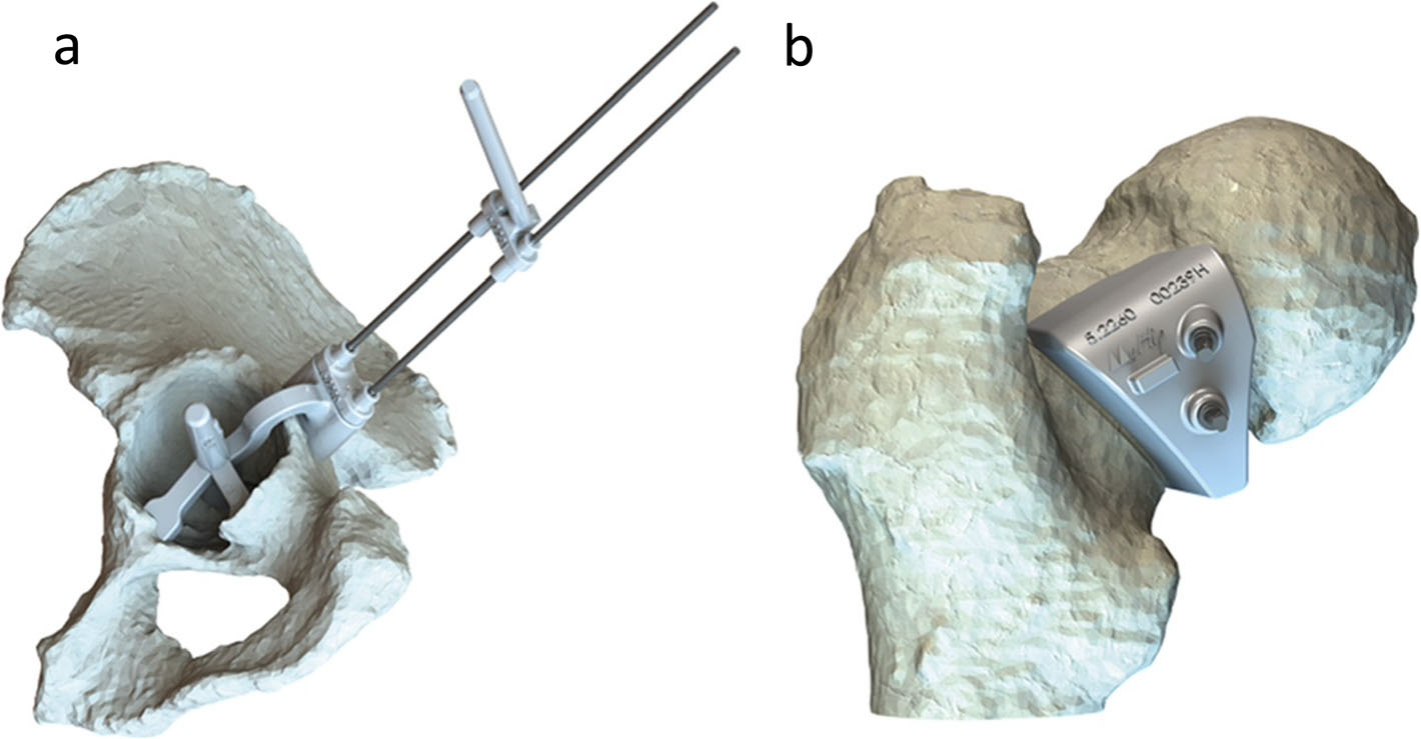
The acetabular and femoral PSI guides. The acetabular guide is seated into the acetabulum, and two pins are inserted through attached drill sleeves. The guide is removed, leaving the two pins to act as either a constrained or unconstrained guide to reaming and component placement (**a**). The femoral guide has a contoured fit to the femoral neck/head and is kept in place for the neck cut by two intraosseous pins (**b**) [13]

During surgery, a patient-specific instrument guide was used to position the cup and cut the femoral neck, Fig. 4.

The acetabular guide was 3D printed to match the acetabular rim. Once seated into the acetabulum two pins were inserted through attached drill sleeves. The guide was then removed, leaving the two pins to act as either a constrained or unconstrained guide to reaming and component placement. The femoral PSI guide was 3D printed to fit the contours of the femoral head-neck junction (ie, no cartilage present at this junction). Once seated, it was secured with two threaded pins. The femoral neck cut was then completed using a standard method, with the saw blade flush on the cutting surface of the guide to deliver a femoral cut at the planned angle and location. The planned cut angle was 45° from the piriformis fossa to the anatomical axis of the femur to match the etched mark on the femoral stem (also 45° to the long axis of the stem). The femoral canal was prepared by the surgeon using the instructions for use provided by the implant manufacturer. The canal was opened using a starter reamer and femoral stem rasps, with sequentially increasing sizes, so that the etched stem marker was level with the cut surface of the femur and the rasp was secure when tested by twisting. The stem was then press-fitted and tested by twisting the implant within the femur and confirming that this did not cause movement between the stem and the bone.

### Post-operative radiological evaluation

All patients underwent conventional standing anteroposterior (AP) radiographs of the pelvis and hip post-operatively. The surgeon assessed size and position of cup and stem as well as restoration of leg length. Number of dislocations and revisions were recorded.

### Statistical analysis

Statistical analysis was performed using Prism 7 (GraphPad Software, San Diego, CA, USA). Paired t-test test was used to determine the difference between the two groups. The reproducibility of planned and implanted cup and stem sizes was determined using the Pearson’s correlation coefficient. The level of significance for all statistical analyses was *p* < 0.05.

## Results

### Feasibility study

Stem and cup components used were within 1 size of the planned. This enabled us to safely optimize the size of the inventory for the pilot study. The feasibility study created the rules for a safe reduction in implant inventory: 1) the planned size of stem, plus one size above and below, and duplicated for all neck options; 2) the planned size, plus one size above and below, of cup, cup liners (both polyethylene and polyethylene hooded), and femoral heads (all 4 neck lengths for both 32 and 36 mm because cups cross boundary between 32 and 36 mm).

### Pilot study

The pilot study showed: 1) 3D surgical planning correctly predicted sizes in 93% (42/45) of the femoral components and 89% (40/45) of the acetabular components; 2) a reduction in the implant inventory size from 101 to 39 components (a 61% reduction in inventory size); 3) good surgical outcomes with no difference between control and experimental groups and importantly, all patients in the group with a reduced inventory size had appropriately sized implants.

### Accuracy of 3D-CT planning

**Fig. 5:**
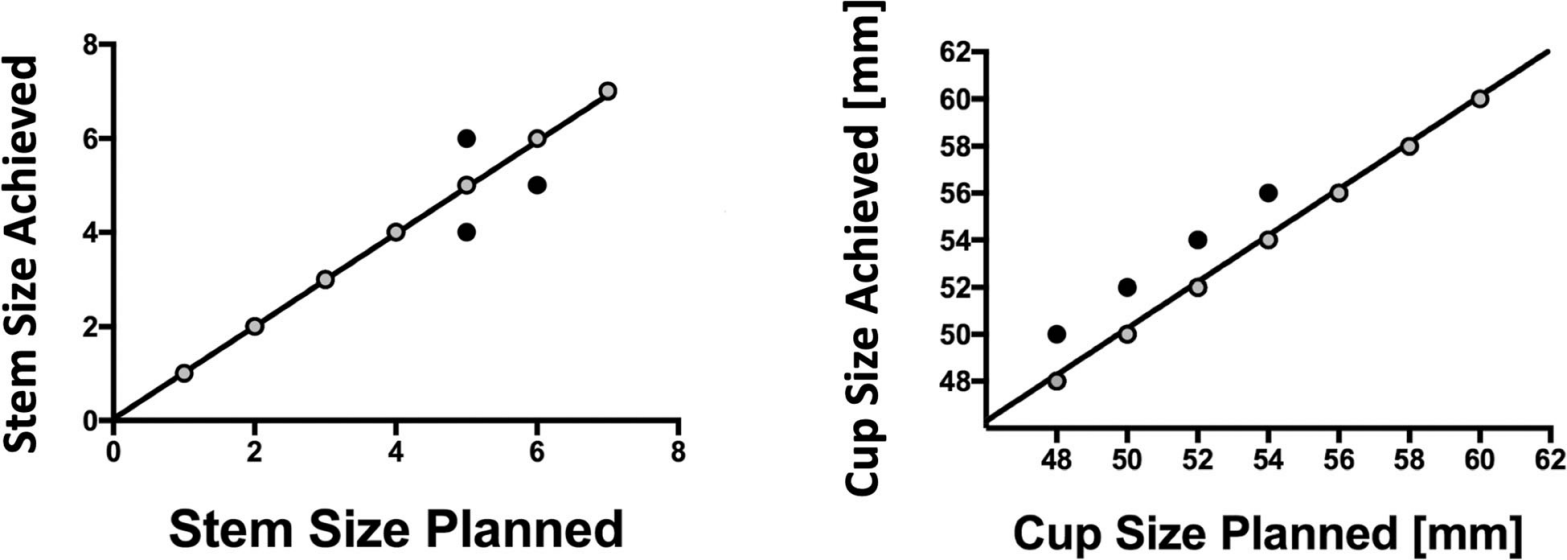
Correlation between the planned values and those achieved for the size of the femoral stem components (*n* = 45); (*r* = 0.99, *p* < 0.0001) (**left image**). Correlation between the planned values and those achieved for the size of the acetabular cups (*n* = 45) (*r* = 0.98, *p* < 0.0001) (**right image**)

All 45 patients had a cup or stem size within one size of planned. The achieved stem size corresponded to the preoperatively planned size in 93% (42/45) of cases. There was a high correlation between the planned and achieved stem sizes (*r* = 0.99, *p* < 0.0001). CT planning correctly predicted size in 89% (40/45) of the acetabular cup components. The 5 discrepancies had cups that were undersized by one size when compared to the planned size. There was a high correlation between the planned and achieved cup sizes (*r* = 0.98, *p* < 0.0001), Fig. 5**.**

**Fig. 6:**
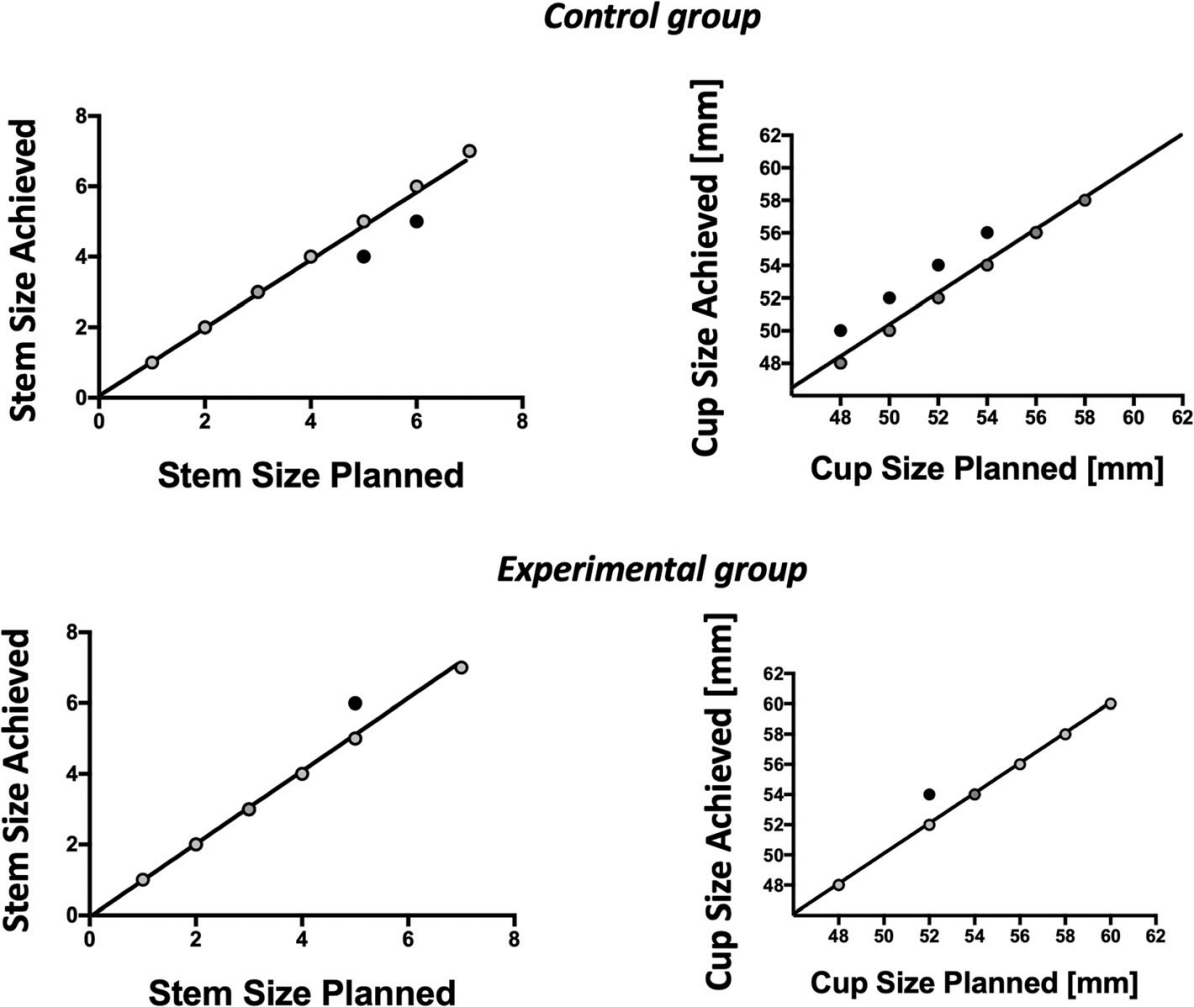
Correlation between the planned values and those achieved for the size of the femoral stem and acetabular cup component for the control (*n* = 25) and experimental (*n* = 20) groups

Accuracy of pre-operative CT planning increased over time, Fig. 6**.**

**Fig. 7:**
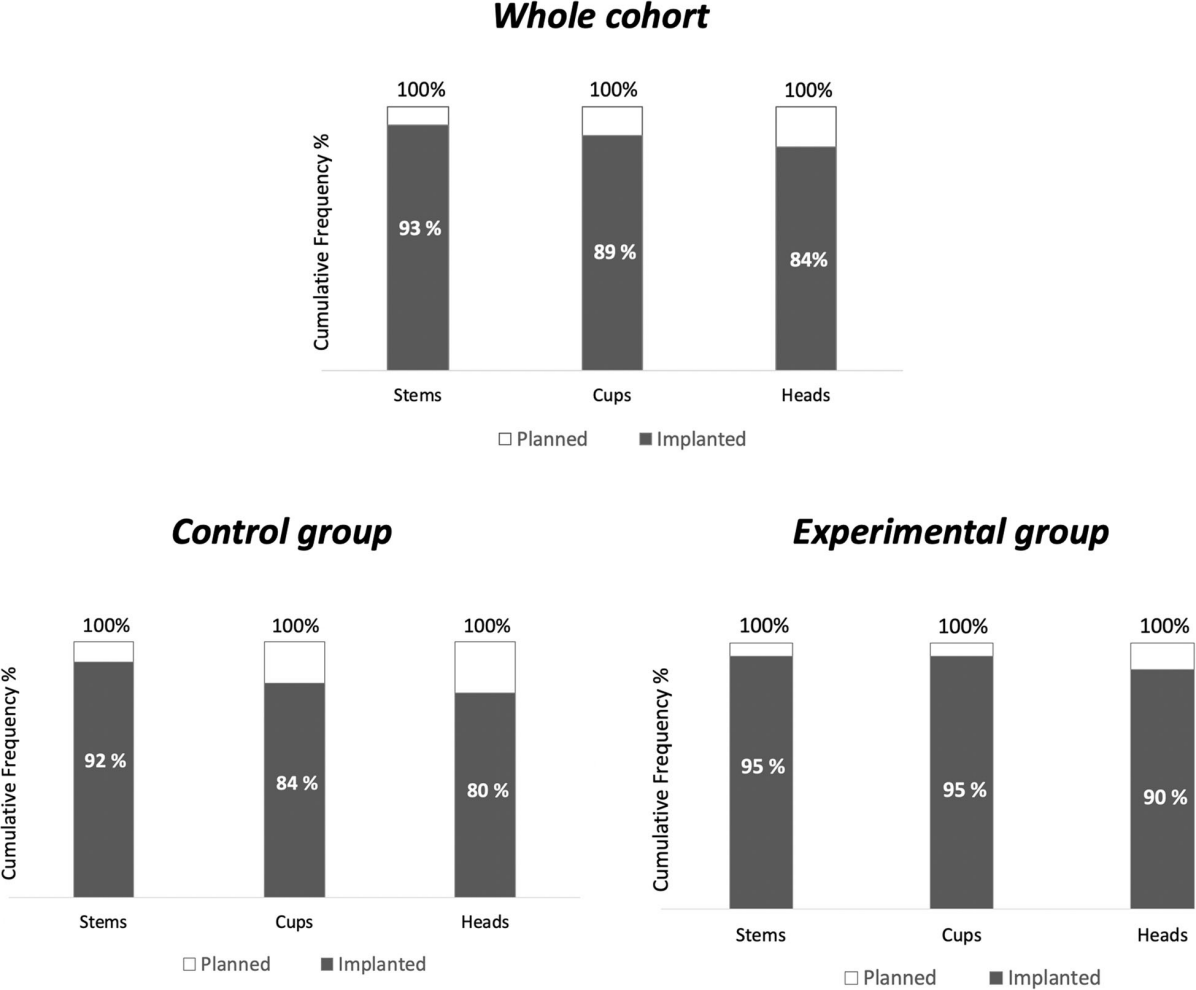
Cumulative frequency of planned size being within ±1 size of target value for the femoral stems, − 1 size of target value for the acetabular cups and ± 2 sizes for the femoral heads in both control and experimental groups

Head diameter was accurately determined in the majority of the cases (80 to 90%), Fig. 7.

### Inventory assessment outcome

No femoral fracture occurred during surgery and there were no errors attributed to a missing implant.

**Fig. 8:**
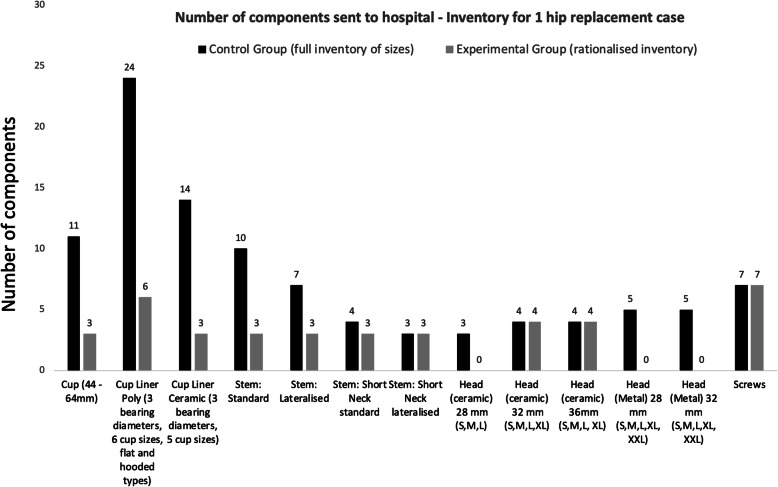
Column plot showing the difference in number of components available for one conventional hip replacement and the sizes needed following 3D-CT planning (comparison between the control and the experimental group)

The full inventory contained 101 implants for the Quadra stem and Mpact Cup system. This was reduced to 39 implants following the feasibility study: a two third reduction (61%), Fig. 8**.**

### Surgical outcome

**Fig. 9:**
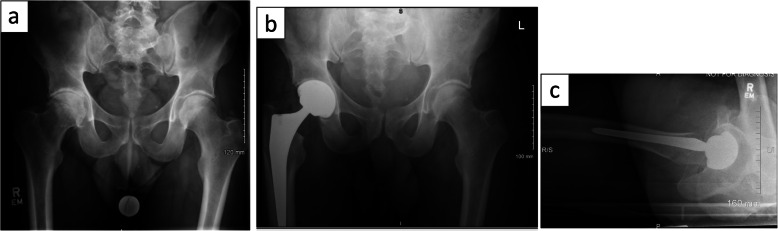
**a** Pre-operative AP radiograph and **b**, **c** post-operative radiographic AP and lateral views of the patient in standing and sitting position showing a satisfactory alignment of the prosthesis

The post-operative course was uneventful for all patients (*n* = 45). Standard post-op 2D radiography showed satisfactory restoration of leg length and femoral offsets Fig. 9**.**

There was one revision operation. This was for cup loosening following a previous acetabular fracture in a patient with protrusio and was not related to the implant size. There were no other revision operations for any cause.

There were two dislocations, which occurred as a result of excessive range of motion: deep hip flexion at 5 weeks post operative; and a deep “child’s pose” during yoga at 12 months post operative. Both of these cases were treated with one closed reduction procedure for each, post reduction CT showed a satisfactory position, and clinical outcomes were good (both had an OHS of 48 out 48 with no further dislocations at last review, which was 12 months and 4 months post dislocation).

## Discussion

THA is one of the most successful and cost effective medical advances of the last 50 years [22]; the implant is the most expensive supply item for joint replacement [1, 5]. The current increase in demand is driving the rationale for cost savings [1, 4].

The modern approach to THA involves a more targeted treatment relying on the use of advanced image modalities for both diagnosis and treatment. Preoperative 3D surgical planning helps determine the correct implant size [18, 23], component orientation and fixation, achieve restoration of femoral offset and limb length [9, 10]. Moreover it enables the use of customised PSI which is sometimes an incorporated step in specialized planning software [24]. Accurate prediction of implant size during preoperative planning is an important factor for successful reconstruction in THA, to avoid intraoperative or postoperative complications. In addition, the accuracy of size prediction may facilitate inventory cost savings and reduce the risk of opening (and thus wasting) the packaging of an implant of the wrong size [25].

Aim of this study was to better understand the impact of the use of 3D surgical planning and PSI on hip implant inventory. This is the first study to investigate the use of CT-based planning software to optimise implant inventory in THA. We found that implant size prediction by 3D planning was accurate in up to 95% of the cases, which enabled us to safely reduce the inventory size by up to 61%. There were no complications relative to implant size.

3D planning correctly predicted implant size in 93% (42/45) of the femoral components and 89% (40/45) of the acetabular components. Stem and cup variability was within one-size in the feasibility study, which gave us the confidence to optimize the inventory for the pilot study.

The ability of 3D planning to predict implant size was up to 2.5-fold superior to that reported when 2D templating is used (accurate in about a third of cases) [8, 26, 27]. A number of studies on cementless THAs have evaluated the accuracy of the preoperative planning by 2D or 3D templating. The agreement with cup size prediction was found to be between 20 and 42.2% with 2D planning and 86–96% with 3D planning, while the agreement with stem size prediction was 19–68.8% with 2D planning, 52–100% with 3D planning [25, 27].

Our results are in accordance with the existing literature, Table 2.

**Table 2:** Rate of prediction for cup and stem size when using 3D planning

Reference	Subjects n	Surgical Indication	Planning Software	Cemented/C.less Total Hip System	Stem Modularity (Yes/No)	Size Prediction (%) Stem-Cup	
(Viceconti, Lattanzi et al. 2003) [15]	29	DDH (65.6%)	Hip-Op	C.less	Y	51.7	65.5
(Sariali, Mouttet et al. 2009) [17]	223	OA	HIP-PLAN	C.less	Y	94	86
(Huppertz, Radmer et al. 2011) [28]	92	NA	3D-Hip Plan®	99% C.less	Y	NA	NA
(Sariali, Mauprivez et al. 2012) [16]	60	OA	Hip-Plan™	C.less	Y	100	96
(Hassani, Cherix et al. 2014) [18]	50	NA	HIP-PLAN	C.less	Y	100	94
(Inoue, Kabata et al. 2015) [23]	57	DDH	ZedHip	C.less	N	65	92
(Mainard, Barbier et al. 2017) [29]	31	OA	hipEOS™	C.less	N	84^a^	92^a^
(Ogawa, Takao et al. 2018) [25]	111	DDH	CT-Based Hip Navigation System	C.less	N	86	94
(Wako, Nakamura et al. 2018) [30]	46	OA (78%)	ZedHip	C.less	Y	92^a^	90^a^
**Current Study**	45	OA	MyHip	C.less	N	93	89

*DDH* Developmental Dysplasia of the Hip; *NA* Not Available; *OA* Osteoarthritis

^a^: within one size

2D templating is still the most commonly used planning method, despite its lower accuracy when compared to CT planning [15, 16], particularly when using cementless components [31, 32] over cemented implants [9, 33] and the magnifications issues associated with conventional 2D radiographs.

A number of barriers hindrance the widespread use of new technology.

The use of CT data introduces new issues related to how best to display and use all information in order to provide surgeons with a user-friendly interface [34]. Another barrier to large adoption of CT planning is the learning curve associated with new technology and systems. Moreover, CT-based planning platforms that are not tied to an implant manufacturer, have recently been introduced to the European and US marketplace, but their uptake has been limited due to software and imaging costs, radiation dose and a lack of standardized optimized imaging protocols.

The use of 3D surgical planning has been largely limited due to the associated ionizing radiation exposure to the patient, limited availability of generic software planning solutions, high cost and limited availability of pre-operative CT imaging protocols. Recent advances in low-dose CT technology tailored for the specific purpose of planning orthopaedic surgery have made justifying the radiation exposure to the patient considerably easier [21, 28]. The new imaging protocols include imaging of the hip, knee and ankle regions at a comparatively lower effective radiation dose without a significant trade off in image quality [21, 28]. These protocols have seen a four-fold reduction in radiation and can be as low as equivalent to 3 pelvic radiographs [35], enhanced further by the introduction of orthopaedic metal artefact reduction (MARS) sequences on many modern CT scanners, allowing suppression of noise artefacts produced by pre-existing metal implants.

Our results show a reduction in the size (up to 61%) of implant inventory based on the use that is conventionally adopted when the cases are not 3D planned for our single surgeon series.

3D surgical planning aids surgical planning and implant sizing with greater accuracy, crucial for good functional hip reconstruction; it therefore can help minimizing the number of surgical trays used during the operation [15, 36, 37]. 3D planning enables a reduction in intraoperative guesswork, and allows for an optimised implant inventory with the potential to reduce the costs to the both manufacturer and hospital without additional risk [23]. However, it is currently unknown what effect CT planning has on the size of hip implant inventory in hospitals.

As the technological advancements in the field of orthopaedics have led to the introduction and development of sophisticated tools, 3D surgical planning is a promising field in which many more developments can be expected providing a quicker and more accurate surgery and the development of an optimal inventory of implants with benefits for all the parts involved in the chain.

We acknowledge limitations. First, we evaluated only one design of implant for both acetabular and femoral components, therefore our findings relative to the reduction in inventory size cannot be generalized to every 3D planning platform and implant type. They can be used as a baseline comparison for future studies aimed at quantifying inventory size reduction. Secondly, this study was based on a series of cases performed by one experienced consultant orthopaedic surgeon, therefore it is unknown whether similar results can be expected for surgeons with different level of experience and using different surgical approaches.

## Conclusion

Planning of hip arthroplasty surgery on a 3D virtual CT-based model is useful to surgeons to help predict the size of the implants to be used in the operating room. The Medacta MyHip system used in this study can accurately predict component size for the femur and the acetabulum.

Based on the experience with our single surgeon series, there was a considerable reduction in the associated inventory use in the CT planned series vis-à-vis with the non-CT planned cases.

We envisage the potential for a phased introduction of a reduced hip implant inventory, and this should probably result in all sizes between 2-sizes below planned up to 2-sizes above planned.

CT planning may become more widely adopted as both manufacturer and hospital seek to be more cost effective in the delivery of hip arthroplasty. A longer-term study is needed to help determine the degree of generalizability of the findings to the available planning software solutions, surgeons and implant manufacturers.

## Data Availability

The datasets used and/or analysed during the current study are available from the corresponding author on reasonable request.
